# A comparison of running and contact loads in U18 and U20 international rugby union competition

**DOI:** 10.5114/biolsport.2022.112086

**Published:** 2022-01-21

**Authors:** Alexis Peeters, Julien Piscione, Mathieu Lacome, Christopher Carling, Nicolas Babault

**Affiliations:** 1 INSERM UMR1093-CAPS, Université Bourgogne Franche-Comté, UFR des Sciences du Sport, F-21000, Dijon; 2 Performance Department, Fédération Française de Rugby (FFR), Marcoussis, France; 3 University of Evry, University of Paris Saclay, Evry, France; 4 Performance and Analytics Department, Parma Calcio 1913, Parma, Italy; 5 Research Dept, Sport Laboratory, Expertise and Performance (EA 7370), French Institute of Sports (INSEP), Paris, France; 6 Performance Department, Fédération Française de Football (FFF), Paris, France; 7 Centre d’Expertise de la Performance, Université Bourgogne Franche-Comté, UFR des Sciences du Sport, F-21000, Dijon

**Keywords:** GPS, Performance, Youth, Game sequences, Peak activity

## Abstract

The purpose of the present study was to characterize and compare locomotor and contact loads in U18 and U20 international rugby union competition during matches, and specifically during peak match-play phases using short rolling epochs and continuous ball-in-play (BIP) sequences. 20 international matches from French national teams were analysed in the U18 and U20 Six Nations Tournament respectively and World Rugby U20 Championship. Running loads were quantified using global positioning devices (16 Hz) and contact loads via video match analysis software. Players were split into forward (U18, n = 29; U20, n = 32) and back positional groups (U18, n = 20; U20, n = 24). Compared with U20 peers, U18 players covered a higher total distance (effect size (ES) = -0.76 ± 0.25) and at high-speeds per minute (> 4 m · s^-1^; ES = -0.55 ± 0.25) and performed more accelerations (ES = -0.71 ± 0.25). While a greater frequency of BIP sequences > 90 s duration was observed in U20s versus U18s match-play, U18s covered more total distance and high-speed distance (ES = -0.42 ± 0.13 and -0.33 ± 0.13 respectively) per minute during these longer sequences. During peak rolling phases shorter than 4 minutes, no clear differences existed between age categories in running activity, while U20 forwards performed more contact actions than U18 peers. The match-play loads observed in the present international U18 players suggest that they are ready to respond to the overall and peak demands observed in U20 competition. Moreover, the present information on peak activity phases can aid design of overload high-intensity conditioning sessions to respond to the running- and contact-demands identified in those competitions.

## INTRODUCTION

Rugby Union is considered one of the most intense and physically demanding team sport games [1]. At elite senior standards, a comprehensive body of research has quantified the physical characteristics in match-play [2, 3, 4, 5, 6]. Analyses using micro-sensor technology such as Global Positioning Systems (GPS) show that the game is intermittent in nature [7]. Players perform frequent bouts of high-speed running and participate in intense physical collisions such as tackling and static actions including scrums, rucks and mauls [8]. In contrast, less information [9, 10] exists on the demands in younger elite players, notably in international competition and how these potentially evolve across different age categories. A comparison of U18 versus U20 amateur rugby union competition reported greater distances per minute covered by players in the former age category [9]. However, studies of U18 competition have mostly considered English players performing at U18 county or school level [9, 10, 11]. Accordingly, a comparison of external load (locomotor and physical contact events) in international U18 versus U20’s competition would provide some answers on whether participation at U18 standards provides an appropiate platform for preparing players physically for U20’s standards. In addition, limited knowledge on whether movement demands in U20s international match-play are sufficient to prepare players for senior international rugby [12]. Such information could notably help in targeting the specific physical qualities and outputs that younger players need to work on to continue their progression [12].

A substantial number of studies have reported the average game-play demands of rugby union, generally over 80-minutes or across playing halves [4, 13]. Yet given the fluctuating nature of running demands, whole-match averages do not reflect the peak intensities reached intermittently during pivotal phases throughout play. Knowledge of efforts during peak match-play periods provides information to help prepare players physically to face the most demanding requirements of competition; frequently known as ‘worst-case scenario’ phases [14]. Training volume and intensity can be specifically referenced against these peak periods of activity to ensure the desired physical stimulus is reached for both the group as a whole and across playing positions. Two methods are generally used to provide key insights into the more demanding periods of play: physical efforts during short epochs – both fixed and rolling, usually of 1 to 10 mins duration [15, 16], and continuous ball-in-play (BIP) sequences determined according to the entire duration of the sequence [13, 17, 18, 19]. Irrespective of the method employed, these epochs typically demonstrate substantial differences compared to average whole match demands. However, while rugby union is a contact-dominant team sport, to our knowledge, no study has attempted to characterize and compare peak demands for locomotor activity and physical collision events concomitantly, and particularly in international U18 and U20 competition. Such information could be used as a foundation for fashioning isolated physical conditioning drills and small-sided games both generally and for positional groups specifically in younger categories.

The propose of the present study was to characterize and compare locomotor and contact loading in U18 and U20 international rugby union competition for the whole-match and during phases of peak match-play activity including both continuous BIP sequences and short rolling epochs. Accounting for findings in a review by Till et al. [20], showing that younger players perform higher running loads at school or academy level, it was hypothesized that even at international level U18 players would perform more locomotor activity than U20 peers whereas the latter would perform more contact-events [20].

## MATERIALS AND METHODS

### Experimental Approach

External loads represented by both running activity and physical contact events were investigated in international French U18 and U20 youth rugby union players during official match-play using GPS and video match analysis software (SportsCode). Data were collected in 20 international matches: the U18’s played 9 matches during the U18 Six Nations Tournament (7 matches) and Aon^®^ U18 International Series (2 matches) while the U20’s played a total of 11 matches of which 9 were in the U20 Six Nations Tournament and 2 in the World Rugby U20 Championship. Data were collected in matches in these tournaments across three successive seasons: 2016/2017, 2017/2018 and 2018/2019. Running and contact loads were analysed over the whole-match, and specifically during peak match-play phases including short rolling epochs and continuous ball-in-play sequences.

### Participants

A total of 105 players participated (U18, n = 49; U20, n = 56) in the present study. These were split into forward (U18, n = 29; U20, n = 32) and back positional groups (U18, n = 20; U20, n = 24). Participants’ age and anthropometric characteristics are reported in Table 1.

**TABLE 1:** Age and anthropometric characteristics of U18 and U20 players.

	U18	U20
**Forwards**		
**N**	29	32
**Age (y)**	17.8 ± 0.5	19.5 ± 0.6
**Stature (cm)**	187.0 ± 8.2	190.6 ± 6.7
**Body mass (kg)**	104.2 ± 11.1	110.7 ± 9.6
**Match observations**	44	68

**Backs**		
**N**	20	24
**Age (y)**	18.0 ± 0.3	19.5 ± 0.7
**Stature (cm)**	178.9 ± 4.6	180.7 ± 6.4
**Body mass (kg)**	82.6 ± 7.9	85.2 ± 6.8
**Match observations**	28	45

Note: Data are presented as mean ± SD.

To ensure player confidentiality, all performance data were anonymized before the analysis. Prior to participation, all the players received comprehensive verbal and written explanations of the study. While data arose as a condition of the players’ participation in their respective national teams whereby they underwent daily monitoring during training camps and competition, written informed consent to participate was obtained, with a parent or guardian providing this for all players under 18 years, in conformity with the recommendations of the Declaration of Helsinki. Permission for the study was also obtained from the scientific research board of the French Rugby Federation.

### Data collection procedures

### Running load

Players wore a portable GPS tracking device (SensorEverywhere V3, Digital Simulation, France), sampling at 16 Hz. The GPS system was placed in a customized pocket in their playing shirt and located between the scapulae. To limit potential inter-unit variability, each player wore the same unit for the entire duration of each competition. Thirty minutes prior to the start of each match, the GPS units were activated to ensure clear satellite reception. Data were captured and computed using propriety SensorEverywhere Analyser software (Digital Simulation, Paris, France). Data were excluded if one of the following criteria was met: number of satellites < 6, horizontal dilution of precision (HDOP) > 2.3 and visual inspection of raw traces of velocity report irregularities [21]. The mean number of satellites and HDOP during match play were 10 ± 2 and 1.7 ± 0.4 respectively. Recent research assessing the inter-unit reliability of the SensorEverywhere GPS devices demonstrated a trivial difference for typical error of measurement and a small difference for maximal sprinting speed (0.5 ± 0.1%) and maximal acceleration (3.9 ± 0.6%) respectively [22].

### Contact load

A trained operator coded physical contact-related match actions including tackles, rucks, collisions (when the player carries the ball into the contact), mauls and scrums using video analysis software (SportsCode Elite, Version 10, Hudl, USA). Active participation in a scrum was considered to be from the front row engagement to break up or when the player was seen to be detached following the release of the ball [6]. Backrow players were removed from the scrum coding. Active participation in periods of rucking and mauling was timed from when a player’s shoulder entered into contact with the ruck or maul to their detachment from the event [8]. Tackles were considered as actions when a player physically attempted to stop a ball carrier whilst on their feet [8]. Collision events were counted when a physical contact was made between an attacker with a player in the defensive line [23].

The operator also used the video-analysis software to quantify ball-in-play time (effective playing time) as this contextual factor influences time-related changes in running and skill-related match performance in team sports [24]. Contextual variables relating to the matches are presented in Supplementary Table 1.

### Data Processing

To synchronize running- and contact-load data, a timestamp marker was created in both GPS and video analysis software at kick-off (when the ball hit the ground). Physical contact-related data was subsequently imported from the Sportscode software into the propriety GPS software.

Three arbitrary running intensity zones were used to adjust running-load data measured in meters (m): total distance (TD), high-speed distance (HSD, velocity > 4 m · s^-1^) and very-high speed distance (VHSD, velocity > 7 m · s^-1^) [25, 26]. Running load was also assessed using the total number of accelerations performed (actions > 2.5 m · s^-2^) [27]. Finally, information on the concomitant number of physical contacts was also provided.

Running-data and contact events were used to determine match demands and compare loads in U18 and U20 players using three approaches:

1) Averaged external match load profiles (data were only included if players completed a minimum of one match half).
2) Ball-in-play (BIP) sequence analysis: the frequency of occurrence of BIP sequences for durations lasting less than 30 s, between 30 to 60 s, 60 to 90 s, 90 to 120 s, and over 120 s was determined. Concomitant running and contact data for BIP sequences lasting more than 90 s are presented. Data for all the other sequences are provided in the supplementary tables except for very short sequences (< 30 s) which were discarded in order to avoid a skewed representation of intensity [17].
3) Peak activity periods: total distance and operator coded contact-events were used singly to define the peak locomotor and contact periods during peak activity periods. A rolling average of 30 s and 1, 2, 3, 4, 5, 6, 8, 10, 12 and 15 min with a step of 1/16 s was used [18].

### Statistical Analysis

Data are presented as means ± standard deviations (SD). A magnitude-based decision approach to the statistical analysis was adopted [28, 29]. Comparisons of U18 and U20 performance and across playing positions were calculated with 90% confidence intervals (90% CI) using specifically designed Excel spreadsheets [30]. Quantitative chances of greater or smaller changes in performance variables were assessed qualitatively and reported as follows: 75–97.5%, likely; 97.5–99%, very likely; > 99%, almost certain [31]. Effect sizes (ES) were quantified to indicate the practical meaningfulness of the differences in mean values. The ES was classified as trivial (< 0.2), small (> 0.2–0.6), moderate (> 0.6–1.2), large (> 1.2–2.0) and very large (> 2.0–4.0) [28]. If the 90% CI overlapped positive and negative values, the magnitude was deemed unclear. The chances that the changes in running or contact load were greater for a group (i.e. greater than the smallest worthwhile change, SWC [0.2 multiplied by the between-subject standard deviation, based on Cohen’s d principle], similar or smaller than the other group, were calculated.

## RESULTS

### General match activity profile

Results (Table 2) show that collectively U18 players covered more TD and HSD and performed more accelerations than U20 peers. The TD and HSD were almost certainly higher in U18 forwards compared with U20 forwards. The VHSD was possibly higher in U18 forwards. Backs belonging to the U18s demonstrated higher values for TD, HSD and accelerations than U20 peers. Unclear differences for VHSD were observed for U18 versus U20 backs. Differences in the frequency of contacts were unclear between U18 and U20 levels irrespective of playing position.

**TABLE 2:** Comparisons of running- and contact-loads performance per minute of play between U18 vs U20 players for the team collectively and across back and forward positions.

				**U20 vs U18**	
**All**	**U18**	**U20**	**%Diff 90%CI**	**ES** ± **90%CI**	**%Chance**
**% Ball In Play**	38.6 ± 3.2%	38.7 ± 4.5%	0 ± 8	0.02 ± 0.74	34/35/31
**TD (m · min^-1^)**	74.3 ± 7.5	68.4 ± 7	-7 ± 2	-0.76 ± 0.25	0/0/100
**HSD (m · min^-1^)**	12.0 ± 5.9	9.3 ± 4.3	-23 ± 10	-0.55 ± 0.25	0/1/99
**VHSD (m · min^-1^)**	0.6 ± 0.7	0.5 ± 0.7	-8 ± 31	-0.07 ± 0.25	4/77/19
**Total Acc (n · min^-1^)**	0.5 ± 0.2	0.4 ± 0.1	-23 ± 8	-0.71 ± 0.25	0/0/100
**Contact (n · min^-1^)**	0.4 ± 0.2	0.4 ± 0.2	-1 ± 13	-0.02 ± 0.25	8/80/12

			**U20 vs U18**		
**Forwards**	**U18**	**U20**	**%Diff 90%CI**	**ES** ± **90%CI**	**%Chance**
**TD (m · min^-1^)**	71.6 ± 5.6	65.4 ± 4.7	-9 ± 2	-1.21 ± 0.32	0/0/100
**HSD (m · min^-1^)**	9.3 ± 4.4	6.6 ± 2.6	-29 ± 12	-0.80 ± 0.32	0/0/100
**VHSD (m · min^-1^)**	0.3 ± 0.4	0.2 ± 0.3	-41 ± 43	-0.30 ± 0.32	1/30/69
**Total Acc (n · min^-1^)**	0.4 ± 0.2	0.3 ± 0.1	-23 ± 11	-0.62 ± 0.32	0/2/98
**Contact (n · min^-1^)**	0.6 ± 0.2	0.6 ± 0.1	0 ± 8	-0.01 ± 0.32	15/67/18

			**U20 vs U18**		
**Backs**	**U18**	**U20**	**%Diff 90%CI**	**ES** ± **90%CI**	**%Chance**
**TD (m · min^-1^)**	78.6 ± 8.3	74.0 ± 6.8	-6 ± 4	-0.62 ± 0.40	0/5/95
**HSD (m · min^-1^)**	16.3 ± 5.3	13.4 ± 2.8	-18 ± 10	-0.73 ± 0.40	0/3/97
**VHSD (m · min^-1^)**	1.1 ± 0.9	1.1 ± 0.8	2 ± 30	0.03 ± 0.40	25/57/18
**Total Acc (n · min^-1^)**	0.6 ± 0.1	0.4 ± 0.1	-24 ± 8	-1.12 ± 0.40	0/0/100
**Contact (n · min^-1^)**	0.2 ± 0.1	0.2 ± 0.1	1 ± 18	0.03 ± 0.40	26/56/18


Note: Data are presented as mean ± SD. Abbreviations: TD, total distance; HSD, high-speed distance; VHSD, very high-speed distance; Total Acc, total accelerations; ES, effect size; CI, confidence interval.

No clear difference was observed for the percentage of effective match playing time between the two age groups.

### Ball-in-play sequence analysis

**FIG. 1:**
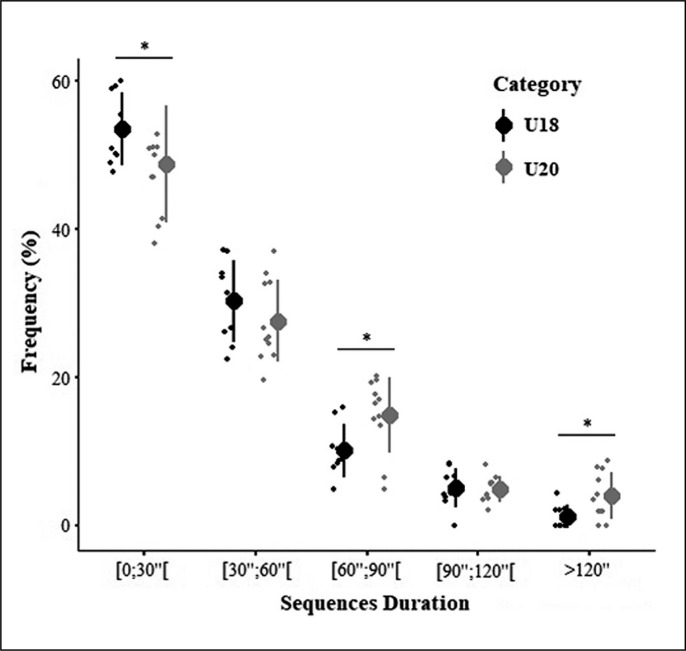
Frequency distribution comparison of BIP sequences between U18 vs U20 matches. Note: *: Likely difference.

In U18s matches, over half of the BIP sequences (Figure 1) lasted less than 30 seconds which was likely more than values observed in U20’s competition (53.5 ± 4.9% vs 48.8 ± 7.9%, ES = -0.67 ± 0.74). Analysis of longer sequences [60 s;90 s[ showed a reversal distribution as likely more sequences were observed in U20 compared to U18 match-play (ES = 1.04 ± 0.74). No differences in the frequency of [90 s;120 s[ sequences were observed between age groups. Likely more sequences of > 120 s duration were reported in U20 matches (ES = 1.02 ± 0.74).

Table 3 reports relative running- and contact-loads during BIP sequences of > 90 s duration in U18 versus U20 play. Owing to a low frequency of > 120 s sequences, these were pooled with sequences lasting [90 s;120 s[. Collectively, U18 players performed more TD and HSD than U20 peers during these sequences. U18’s backs performed more accelerations than U20s peers, while U20 backs covered more VHSD than U18 backs. U20 forwards performed more contact actions than U18 peers. Outcomes for all other performance variables during > 90 s sequences presented trivial or unclear differences. Performance in the other BIP sequences are reported in the supplementary tables.

**TABLE 3:** Comparisons of running- and contact-loads performance per minute during BIP sequences of > 90 s between U18 vs U20 players for the team collectively and across back and forward positions.

			**U20 vs U18**		
**All**	**U18**	**U20**	**%Diff 90%CI**	**ES** ± **90%CI**	**%Chance**
**Duration (min)**	1.8 ± 0.2	2.1 ± 0.5	14 ± 3	0.63 ± 0.13	100/0/0
**TD (m · min^-1^)**	110.8 ± 30.7	99.4 ± 25.5	-10 ± 3	-0.42 ± 0.13	0/0/100
**HSD (m · min^-1^)**	24.1 ± 20.0	17.9 ± 17.5	-25 ± 10	-0.33 ± 0.13	0/5/95
**VHSD (m · min^-1^)**	0.4 ± 2.0	0.8 ± 3.8	105 ± 104	0.13 ± 0.13	12/88/0
**Total Acc (n · min^-1^)**	0.9 ± 0.7	0.7 ± 0.6	-16 ± 10	-0.20 ± 0.13	0/48/52
**Contact (n · min^-1^)**	0.9 ± 0.7	1.0 ± 0.8	15 ± 10	0.18 ± 0.13	38/62/0

			**U20 vs U18**		
**Forwards**	**U18**	**U20**	**%Diff 90%CI**	**ES** ± **90%CI**	**%Chance**
**TD (m · min^-1^)**	107.8 ± 26.2	96.0 ± 22.0	-11 ± 4	-0.50 ± 0.17	0/0/100
**HSD (m · min^-1^)**	17.8 ± 14.3	10.7 ± 12.0	-40 ± 12	-0.55 ± 0.17	0/0/100
**VHSD (m · min^-1^)**	0.4 ± 2.2	0.1 ± 1.2	-73 ± 64	-0.19 ± 0.16	0/54/46
**Total Acc (n · min^-1^)**	0.8 ± 0.7	0.7 ± 0.6	-9 ± 14	-0.10 ± 0.17	0/83/17
**Contact (n · min^-1^)**	1.2 ± 0.7	1.4 ± 0.7	22 ± 10	0.35 ± 0.17	94/6/0

			**U20 vs U18**		
**Backs**	**U18**	**U20**	**%Diff 90%CI**	**ES** ± **90%CI**	**%Chance**
**TD (m · min^-1^)**	115.2 ± 35.9	103.4 ± 28.6	-10 ± 5	-0.38 ± 0.19	0/8/92
**HSD (m · min^-1^)**	33.2 ± 23.3	26.3 ± 19.2	-21 ± 12	-0.33 ± 0.19	0/14/86
**VHSD (m · min^-1^)**	0.4 ± 1.8	1.6 ± 5.3	316 ± 222	0.27 ± 0.19	81/19/0
**Total Acc (n · min^-1^)**	1.0 ± 0.7	0.8 ± 0.7	-24 ± 12	-0.38 ± 0.19	0/7/93
**Contact (n · min^-1^)**	0.6 ± 0.6	0.6 ± 0.5	11 ± 18	0.12 ± 0.19	25/75/0


Data are presented as mean ± SD. Abbreviations: TD, total distance; HSD, high-speed distance; VHSD, very high-speed distance; Total Acc, total accelerations; ES, effect size; CI, confidence interval.

### Analysis of peak activity periods

**FIG. 2:**
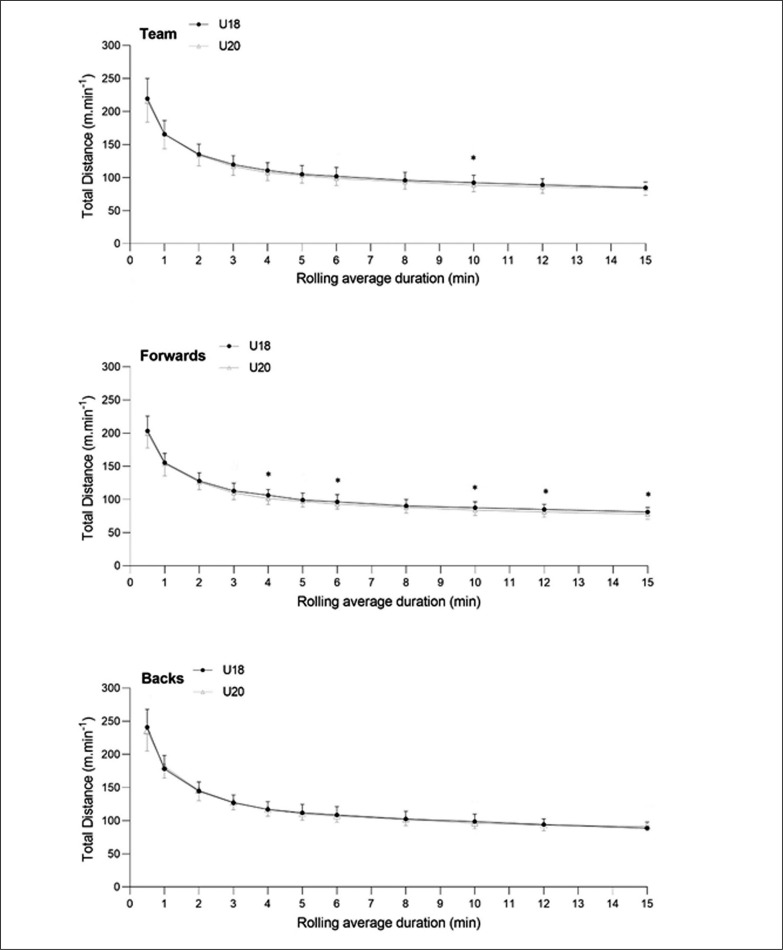
Comparison of maximal locomotor activity associated with peak TD periods between forwards and backs at U18 and U20 levels. Note: *: Likely difference.

No difference was observed between the two age groups in running-load during any of the short peak activity periods for the players as a whole (Figure 2). U18 forwards covered likely more total distance (small ES) than U20 forwards in 4, 6, 10, 12 and 15-min peak TD periods. Regarding backs, unclear differences were observed across age groups during peak periods.

**FIG. 3:**
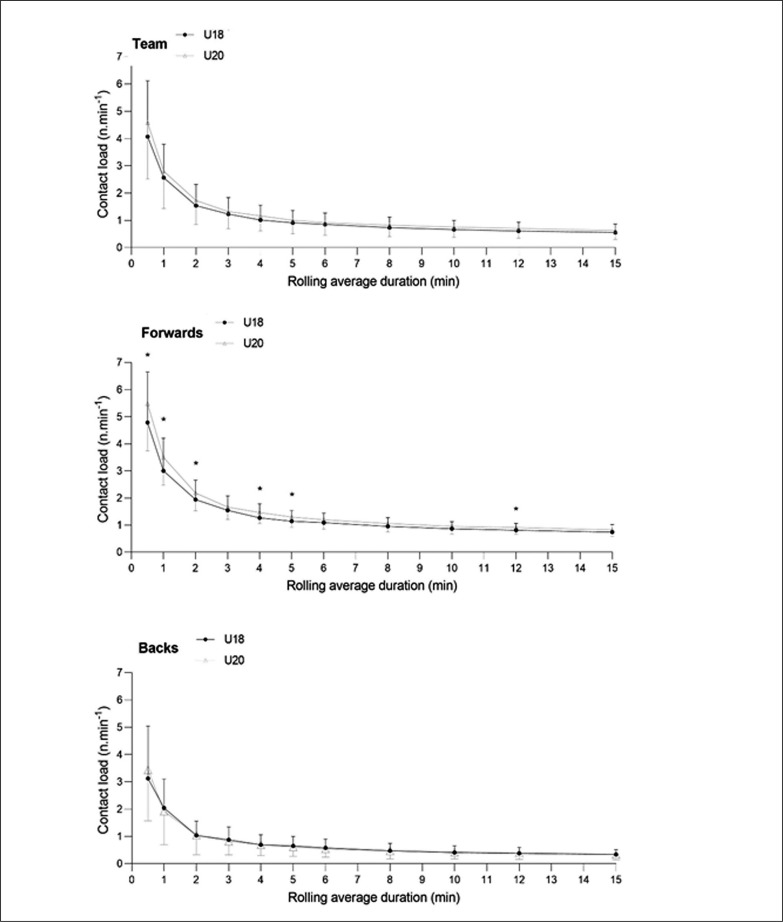
Comparison of maximal number of contacts associated to peak contact-load periods between forwards and backs at U18 and U20 levels. *: Likely difference.

U20 forwards performed likely more contact actions (small ES) than U18 forwards during 30 s, 1, 2, 4, 5 and 12-min peak contact load periods (Figure 3) while no differences were observed in U18 versus U20 backs.

## DISCUSSION

The aim of this study was to characterize and compare running and contact loads in U18 and U20 international rugby union competition. Key findings were: (1) U18 players covered more TD, HSD and performed more accelerations per minute compared with U20 peers, (2) a greater frequency of ball-in-play (BIP) sequences of long duration (> 90 s) was observed in U20 versus U18 match-play, while U18s covered slightly more TD and HSD per minute during these > 90 s sequences and, (3) while no clear differences existed between age categories in running performance during short peak periods of activity, a higher frequency of contacts was observed in U20 forwards.

### General match activity profile

To our knowledge, this study is the first to report running and contact load characteristcs in U18 players in international standard competition and compare with these with data gathered in international U20 peers. The values for running activity observed in both age categories (Table 2), were in the upper range of those reported in previous research on elite standards of play; TD covered per minute ranged from 65 and 74 m · min^-1^ for the present U20 forwards and backs respectively vs 62 and 69 m · min^-1^ observed in other international U20 players [12]. At elite academy U18 club level [10], TD values per minute of 71 and 74 m · min^-1^ were reported for forwards and backs respectively compared with 72 and 79 m · min^-1^ in the present study.

The TD, HSD, and frequency of accelerations were greater in the U18 versus the U20 players. In addition, the VHSD was possibly greater for U18 forwards compared with U20 forwards while an unclear difference was observed between backs. These results tend to confirm trends previously reported across age groups in other elite and sub-elite rugby union populations [9, 11, 12, 32]. Till et al. [20] reported higher relative distances covered by U16 county players versus U20 international players. In addition, HSD and the frequency of acceleration actions are greater in U18 games compared with U20 [9, 11, 12]. Taken together, these findings suggest that running activity per se is not a limiting performance criterion in elite and international rugby union, irrespective of playing position, when graduating through different age categories. Owing to comparable TD covered here and data previously reported in a study comparing U20 and senior international players [12], the present findings also support the idea that international U20 competition is an adequate ‘stepping stone’ for preparing players physically for the overall running demands reported at senior international standards.

In contrast to running activities, no differences regarding the frequency of contact actions were observed across the present age groups, either collectively or for positional role. This result differs to previous findings in county level match-play where a greater frequency of collisions were observed in U16 versus U20 players [9]. This discrepancy across findings could potentially be linked to the French Rugby Federation’s national tactical and technical youth technical development performance plan, where a similar and consistent training plan is implemented across age groups. Similar research is nevertheless warranted in other international populations to verify whether these contact demands reflect those observed at senior standards. Moreover, we can suppose that as body mass increases with age, the magnitude of contact increases although research is necessary to confirm this suggestion.

### Ball-in-play sequences

To our knowledge, this study is also the first to quantify the frequency of occurrence of BIP game sequences and associated physical outputs in U18 and U20 international rugby union players. Similar to Read et al’s [19] findings showed in U18s competition, over half of BIP sequences observed were of < 30 s duration. In contrast, U20s completed a moderately greater number of longer sequences (60–90; and > 120 s). While complementary research using video- and match-analyses is necessary, a reasonable explanation for this difference could be linked to a greater technical and tactical ability in U20 players enabling them to collectively conserve ball possession over longer periods [9].

Regarding the concomitant physical characteristics observed during sequences of play of > 90 s in duration (Table 3), U18s covered almost certainly more relative TD and likely more HSR compared to U20 peers. It is also noteworthy that the TD covered by the present U18s was higher than values observed in senior international rugby union match-play during sequences of the same duration [17]. These results once again tend to imply that running activity per se is not a discriminant factor when progressing through U18 and U20 international age categories and that match demands at younger levels provide adequate opportunities to prepare players for senior international rugby.

While U18s as a whole performed more relative TD during longer playing sequences, contrasting high-intensity demands were apparent across positional groups within the two age groups. The frequency of contact events during these long sequences was approximately 20% higher (small ES) in forwards in U20’s versus U18’s match-play while U20 backs covered more VHSD (small ES) compared to U18s peers. These results suggested that at U20 standards, greater emphasis could be placed on developing position-specific physical skills, through adapted physical conditioning programmes to respond to the high intensity demands occuring in longer sequences of play that occur frequently in this age category.

### Peak Activity Periods

The analysis of collective peak running-load activity (distance run per minute) reported no differences between the two age groups during any of the peak activity periods (Figure 2). The positional group demands observed during a 2-min maximal running activity window showed that performance was not dissimilar to that previously reported in forwards at U18 English academy level [18]. During a 2-min maximal window, English academy forwards covered distances ranging from 121 to 132 m · min^-1^ compared to 128 and 126 m · min^-1^ in the present U18 forwards and U20 forwards, respectively. In contrast, English academy U18 backs in the same study tended to cover lower distances than the present international U18 backs during a 2-min peak activity period (range: 133 to 146 m · min^-1^ versus 144 and 145 m · min^-1^ for the present U18 backs and U20 backs respectively). In comparison to international senior standards, the current results across both positional groups and age categories were comparable for short periods of peak activity (from 1-min to 5-min) [15, 16]. During longer peak activity periods (> 10-min) the present younger players completed greater relative distances than senior international players [16]. To summarize, peak TD distance activity in U18 and U20 match-play were higher than in peers in elite academies yet tended to be stable across these age categories. Performance was also comparable to that observed in senior international players suggesting that younger international players would be able to respond physically to the running demands observed at the very highest standards of the game.

The peak period running data reported above has potential for application in designing training prescriptions based upon ‘worse-case’ scenarios for physical conditioning sessions [15]. Similarly, contact activity during peak periods also provides practitioners with information on contact loading for prescription and monitoring of physical contact training sessions (Figure 3). While no collective differences were observed between the U18 and U20 categories, U20 forwards performed likely more contact actions than U18 peers during short peak periods lasting less than 4-min in duration (ES range from 0.36 to 0.46). These results suggest that when U18’s graduate to U20 level, there is a need to adapt the training contact dose to enhance their capacity to repeat contact events.

### Limitations

In the current study, only the frequency of contact events was quantified, and future research is warranted to determine the intensity of these events (quantified in G) notably using the accelerometer and gyroscope technologies housed within the GPS. As such, this would enable a more precise evaluation of contact demands between the two age groups as one would expect that contact intensity is greater in U20 competition owing to the larger body mass of U20 players [33, 34]. Additional match analysis research is required to determine the concomitant collective and position-specific tactical and technical demands and peak phases to provide a more holistic understanding of match performance. It is also possible that the national playing style guidelines specific to France’s national youth team development program biased the present findings. Accordingly, additional studies ideally using larger sample sizes (matches and players) and data gathered in populations from other nations are necessary [35].

## CONCLUSIONS

To our knowledge, this study is the first to characterize and compare locomotor and contact loads in international U18 and U20 rugby match-play. It appears that the demands observed at U18 level suffice to help prepare players physically for U20 levels while demands in the latter are comparable to those previously reported in senior international match-play. Future work should focus on determining the concomitant technical and/or tactical aspects of play to provide a more holistic understanding of match performance demands at younger levels.

### Practical applications

– The activity profiles observed in international U18 match-play suggest that players in this age group are sufficiently well-prepared physically for the general running and contact loads and those specific to ball-in-play sequences observed at U20 standards.
– The present information on maximal running intensities can inform running loads for high-intensity conditioning sessions and small-sided games in younger elite players.
– These results can also aid practitioners to manage and individualize contact loads across age categories and positional groups during high-intensity training drills.

## Figures and Tables

**SUPPLEMENTARY TABLE 1:** Contextual variables of matches investigated.

	U18	U20
**Win (n) / Loose (n)**	8 / 1	8 / 3
**Victory % / Loose %**	89% / 11%	73% / 27%
**Opponent possession %**	51.5 ± 7.4	49.4 ± 8.4
**France possession %**	48.5 ± 7.4	50.6 ± 8.4
**Total number of tries**	7.2 ± 2.2	7.4 ± 4.2
**Opponent number of tries**	2.6 ± 1.2	2.7 ± 2.5
**France number of tries**	4.7 ± 1.7	4.6 ± 3.5
**Total number of points**	51.9 ± 12.8	56.9 ± 23.2
**Opponent number of points**	20 ± 7.4	21.5 ± 14.8
**France number of points**	31.9 ± 8.1	35.4 ± 22.1

Note: Data are presented as mean ± SD.

**SUPPLEMENTARY TABLE 2:** Comparisons of running- and contact-loads performance per minute during BIP sequences lasting 30 s to 60 s between U18 vs U20 players for the team collectively and across back and forward positions.

				**U20 vs U18**	
**All**	**U18**	**U20**	**Diff% 90%CI**	**ES** ± **90%CI**	**%Chance**
**Duration (min)**	0.7 ± 0.1	0.7 ± 0.1	2 ± 1	0.08 ± 0.06	0/100/0
**TD (m · min^-1^)**	115.0 ± 36.1	101.5 ± 36.5	-12 ± 2	-0.37 ± 0.06	0/0/100
**HSD (m · min^-1^)**	31.4 ± 32.4	21.6 ± 27	-31 ± 6	-0.33 ± 0.06	0/0/100
**VHSD (m · min^-1^)**	1.9 ± 8.4	1.1 ± 5.6	-42 ± 22	-0.11 ± 0.06	0/99/1
**Total Acc (n · min^-1^)**	1.0 ± 1.2	0.9 ± 1.1	-12 ± 6	-0.11 ± 0.06	0/99/1
**Contact (n · min^-1^)**	0.6 ± 1	0.8 ± 1	36 ± 10	0.22 ± 0.06	70/30/0

				**U20 vs U18**	
**Forwards**	**U18**	**U20**	**Diff% 90%CI**	**ES 90%CI**	**%Chance**
**TD (m · min^-1^)**	112.2 ± 32.4	98.7 ± 32.8	-12 ± 2	-0.41 ± 0.08	0/0/100
**HSD (m · min^-1^)**	24.7 ± 27.4	15.4 ± 21.6	-38 ± 8	-0.38 ± 0.08	0/0/100
**VHSD (m · min^-1^)**	0.6 ± 4.4	0.2 ± 2.2	-67 ± 46	-0.12 ± 0.08	0/95/5
**Total Acc (n · min^-1^)**	0.9 ± 1.1	0.8 ± 1	-14 ± 9	-0.11 ± 0.08	0/96/4
**Contact (n · min^-1^)**	0.8 ± 1.1	1.2 ± 1.1	45 ± 11	0.32 ± 0.08	99/1/0

				**U20 vs U18**	
**Backs**	**U18**	**U20**	**Diff% 90%CI**	**ES 90%CI**	**%Chance**
**TD (m · min^-1^)**	119.0 ± 40.5	104.8 ± 40.2	-12 ± 3	-0.35 ± 0.09	0/0/100
**HSD (m · min^-1^)**	41.1 ± 36.3	29.0 ± 30.7	-30 ± 7	-0.37 ± 0.09	0/0/100
**VHSD (m · min^-1^)**	3.7 ± 11.7	2.1 ± 7.7	-43 ± 23	-0.17 ± 0.09	0/71/29
**Total Acc (n · min^-1^)**	1.2 ± 1.2	1.1 ± 1.2	-13 ± 9	-0.13 ± 0.09	0/88/12
**Contact (n · min^-1^)**	0.4 ± 0.7	0.5 ± 0.8	30 ± 19	0.15 ± 0.09	16/84/0


Note: Data are presented as mean ± SD. Abbreviations: TD, total distance; HSD, high-speed distance; VHSD, very high-speed distance; Total Acc, total accelerations; ES, effect size; CI, confidence interval.

**SUPPLEMENTARY TABLE 3:** Comparisons of running- and contact-loads performance per minute during BIP sequences lasting 60 s to 90 s between U18 vs U20 players for the team collectively and across back and forward positions.

				**U20 vs U18**	
**All**	**U18**	**U20**	**Diff% 90%CI**	**ES** ± **90%CI**	**%Chance**
**Duration (min)**	1.2 ± 0.1	1.2 ± 0.1	-2 ± 1	-0.16 ± 0.09	0/75/25
**TD (m · min^-1^)**	107.6 ± 31.4	101.7 ± 31.6	-5 ± 3	-0.18 ± 0.09	0/60/40
**HSD (m · min^-1^)**	23.4 ± 21.7	21.0 ± 21.0	-11 ± 9	-0.12 ± 0.09	0/93/7
**VHSD (m · min^-1^)**	0.9 ± 3.6	0.9 ± 3.8	-7 ± 38	-0.02 ± 0.09	0/100/0
**Total Acc (n · min^-1^)**	0.9 ± 0.9	0.8 ± 0.8	-15 ± 9	-0.16 ± 0.09	0/72/28
**Contact (n · min^-1^)**	0.8 ± 0.9	1 ± 0.9	21 ± 10	0.19 ± 0.09	43/57/0

				**U20 vs U18**	
**Forwards**	**U18**	**U20**	**Diff% 90%CI**	**ES 90%CI**	**%Chance**
**TD (m · min^-1^)**	105.5 ± 28.3	98.9 ± 26.4	-6 ± 3	-0.25 ± 0.12	0/27/73
**HSD (m · min^-1^)**	18.3 ± 17.2	14.8 ± 15.2	-19 ± 11	-0.22 ± 0.12	0/40/60
**VHSD (m · min^-1^)**	0.4 ± 2.1	0.2 ± 1.9	-40 ± 69	-0.07 ± 0.12	0/95/5
**Total Acc (n · min^-1^)**	0.9 ± 0.9	0.7 ± 0.8	-20 ± 12	-0.21 ± 0.12	0/45/55
**Contact (n · min^-1^)**	1.1 ± 0.9	1.4 ± 0.9	27 ± 11	0.32 ± 0.12	94/6/0

				**U20 vs U18**	
**Backs**	**U18**	**U20**	**Diff% 90%CI**	**ES 90%CI**	**%Chance**
**TD (m · min^-1^)**	110.4 ± 35.1	105.3 ± 36.7	-5 ± 5	-0.14 ± 0.14	0/75/25
**HSD (m · min^-1^)**	30.8 ± 25.1	28.6 ± 24.4	-7 ± 12	-0.09 ± 0.14	0/90/10
**VHSD (m · min^-1^)**	1.7 ± 5	1.6 ± 5.1	-4 ± 43	-0.01 ± 0.14	1/98/2
**Total Acc (n · min^-1^)**	1.0 ± 0.9	0.9 ± 0.9	-11 ± 13	-0.13 ± 0.14	0/80/20
**Contact (n · min^-1^)**	0.5 ± 0.7	0.5 ± 0.6	13 ± 19	0.10 ± 0.14	14/86/0


Note: Data are presented as mean ± SD. Abbreviations: TD, total distance; HSD, high-speed distance; VHSD, very high-speed distance; Total Acc, total accelerations; ES, effect size; CI, confidence interval.
